# Porcine Reproductive and Respiratory Syndrome Virus (PRRSV) Inhibits RNA-Mediated Gene Silencing by Targeting Ago-2

**DOI:** 10.3390/v7102893

**Published:** 2015-10-23

**Authors:** Jing Chen, Xibao Shi, Xiaozhuan Zhang, Li Wang, Jun Luo, Guangxu Xing, Ruiguang Deng, Hong Yang, Jinting Li, Aiping Wang, Gaiping Zhang

**Affiliations:** 1 College of Animal Science and Veterinary Medicine, Jilin University, Changchun 130062, China; chenjing519@126.com; 2 Henan Provincial Key Laboratory of Animal Immunology, Henan Academy of Agricultural Sciences, Zhengzhou 450002, China; shixibao@aliyun.com (X.S.); nongkewangli@163.com (L.W.); luojun593@aliyun.com (J.L.); rgd999@163.com (R.D.); 3 College of Life Sciences, Henan Normal University, Xinxiang 453007, China; zhangxiaozhuan0103@126.com (X.Z.); yanghong@htu.cn (H.Y.); Ljt66882004@126.com (J.L.); 4 Department of Bioengineering, Zhengzhou University, Zhengzhou 450000, China; pingaw@126.com; 5 College of Veterinary Medicine and Animal Science, Henan Agricultural University, Zhengzhou 450002, China

**Keywords:** porcine reproductive and respiratory syndrome virus (PRRSV), RNA silencing, non-structure protein 1α (nsp1α), nsp11, Ago-2, J0101

## Abstract

Porcine reproductive and respiratory syndrome virus (PRRSV) infection strongly modulates the host’s immune response. The RNA silencing pathway is an intracellular innate response to viral infections. However, it is unknown whether PRRSV interacts with cellular RNA silencing to facilitate the viral infection. Here, we report for the first time the interaction between PRRSV and RNA silencing in both the porcine macrophages and African green monkey kidney cell line (MARC-145) cell line, which were derived from African green monkey kidney cells and highly permissive for PRRSV infection. Our data demonstrated that PRRSV suppressed RNA silencing induced by short-hairpin (sh) RNA, double-strand (ds) RNA and microRNA (miRNA) and downregulated the expression of argonaute protein-2 (Ago-2), which is a key protein of the RNA silencing pathway in animal cells. Further, exogenous introduction of siRNA and shRNA downregulated Dicer or Ago-2 proteins of the cellular RNA silencing apparatus in MARC-145 cells and porcine macrophages, which, in turn, increased the viral replication and titers. The viral non-structure protein 1α (nsp-1α) and nsp11 of PRRSV were identified as the suppressors for cellular RNA silencing (RSSs) to downregulate the Ago-2 protein. Our results identify that PRRSV, through its nsp proteins, suppresses the cellular RNA silencing apparatus in favor of viral infection and supports a co-evolutionary process of the virus and the cellular RNA silencing process.

## 1. Introduction

RNA silencing, which could be induced by small interfering RNAs (siRNA) and microRNA (miRNA), is a conserved mechanism for most eukaryotic post-transcriptional gene regulation [1,2]. siRNAs are derived from the cytoplasmic cleavage of the long doubled RNA(dsRNA) into ~21 nt siRNA duplexes by dsRNA-specific endoribonuclease (RNase) Dicer, then through base pairing, one strand of the siRNAs is incorporated into the RNA-induced silencing complex (RISC) and guides RISC to endoribonucleic cleavage or to silence its target mRNA with the essential help of Ago-2. The cellular miRNA genes could encode the endogenous cellular miRNAs [3,4]. Because the structures of short hairpin RNAs (shRNAs) are similar to the structures of pre-miRNAs, shRNAs are used as the precursor molecules of siRNA in cells.

Studies documented that RNA silencing is a natural innate immunity to resist viruses in plants or invertebrates [5,6,7]. During virus infection, the anti-viral activity of RNA silencing could be activated by the emergence of virus-specific dsRNA or miRNA, finally destroying the RNA of the virus. However, during the co-evolutionary process of the virus and the cellular RNA silencing process, the viruses have produced suppressors of RNA silencing (RSSs) to overcome the anti-viral RNA silencing [8,9]. Furthermore, accumulating evidence suggested that RNA silencing might also be an important innate antiviral immunity in vertebrates [5,10,11,12]. Firstly, the RNA silencing pathway in mammal cells is competent. Secondly, small RNAs induced by the virus have been identified in several animal viruses [13]. Thirdly, inactivation of Dicer could influence the replication of animal viruses [9,14,15,16]. Finally, many animal viruses encoded RSSs to inhibit RNA silencing [5].

PRRSV is a small, enveloped positive-stranded RNA virus [17], can make sows reproduce unsuccessfully and cause pigs to have respiratory difficultly or to experience a secondary infection easily, and PRRSV infection has caused serious economic losses in pig farms and factories [18].

PPRSV could induce immunosuppression and form a persistent infection. Previous studies have focused on the innate and adaptive immune responses of PRRSV, but it is unknown whether PRRSV could suppress RNA silencing or not, since the RNA silencing pathway is also an intracellular innate response to viral infection.

Therefore, the aims of the present work are to address whether PRRSV could suppress RNA silencing, whether RNA silencing could take part in controlling PRRSV replication and whether PRRSV could encode RSSs to help the virus overcome the cellular RNA silencing process.

## 2. Results

### 2.1. PRRSV Suppressed the RNA Silencing Induced by shRNA, dsRNA and miRNA

One characteristic of PRRSV was that PRRSV could induce a persistent infection [18], which indicated that PRRSV was able to overcome the cellular antiviral response and further may overcome the RNA silencing. To test this hypothesis, this work investigated whether PRRSV could restore shRNA, dsRNA or miRNA-induced gene silencing.

**Figure 1:**
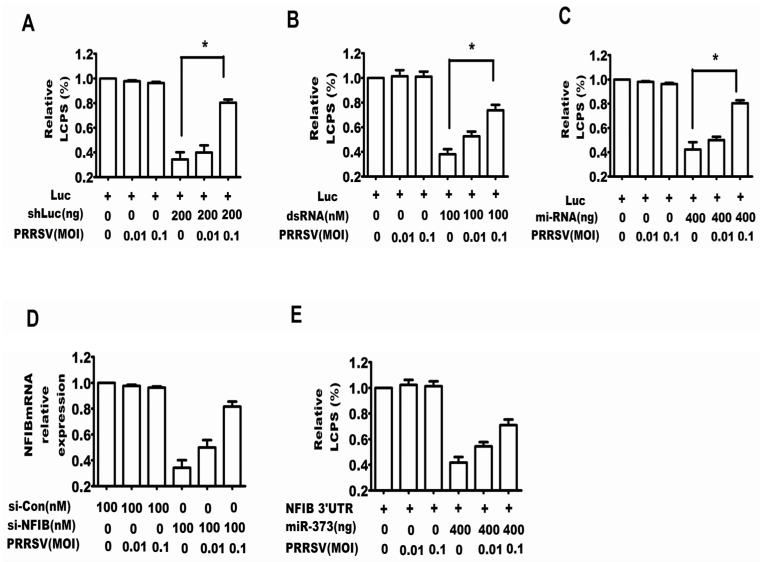
PRRSV inhibited shRNA, dsRNA and miRNA-induced gene silencing. Cells of MARC-145 (African green monkey kidney cell line) were co-transfected with firefly luciferase expression plasmid CMV-Luc, internal control plasmid RLTK and firefly luciferase-specific shRNA (shLuc) (**A**); firefly luciferase-specific dsRNA (dsRNA) (**B**); pcDNA6.2-miR4 and psiCHECK-UL-28 (**C**); NFIB-specific siRNA (si-NFIB) (**D**); pcDNA6.2-miR-373 and psiCHECK2/NFIB 3′ UTR (NFIB 3′ UTR) (**E**). Six hours later, the cells were infected with PRRSV at an MOI of 0, 0.01 or 0.1. Additionally, 48 hour later, the cells were ready for the dual luciferase reporter assay. The experiments were repeated three times. The results shown are from one of these with similar observations. * *p* < 0.05.

Firstly, we tested whether PRRSV was capable of restoring the silencing of the luciferase gene induced by shRNA. Figure 1A shows that the shLuc plasmid strongly decreased the expression level of luciferase, while PRRSV infection could inhibit the shRNA-induced silencing of luciferase.

Then, it was tested whether PRRSV was capable of restoring the silencing of a luciferase gene induced by dsRNA, and the method was similar to that of the assay for shRNA. Figure 1B shows that dsRNA strongly decreased the expression level of luciferase, while PRRSV infection also inhibited the shRNA-induced silencing of luciferase.

A previous study has shown that miR4 of the Marek’s disease virus targeted the viral mRNA UL-28 [19]. Therefore, we explored whether PRRSV also inhibited the miRNA-induced gene silencing. Figure 1C shows that the infection of PRRSV could inhibit the miRNA-induced silencing of luciferase. To confirm the above results, the endogenous gene NFIB was selected to perform a similar experiment, and the results in Figure 1D and E show that PRRSV also inhibited the si-NFIB- and miR-373-induced silencing of NFIB.

### 2.2. Dicer and Ago-2 Are Involved in Protection against PRRSV

**Figure 2:**
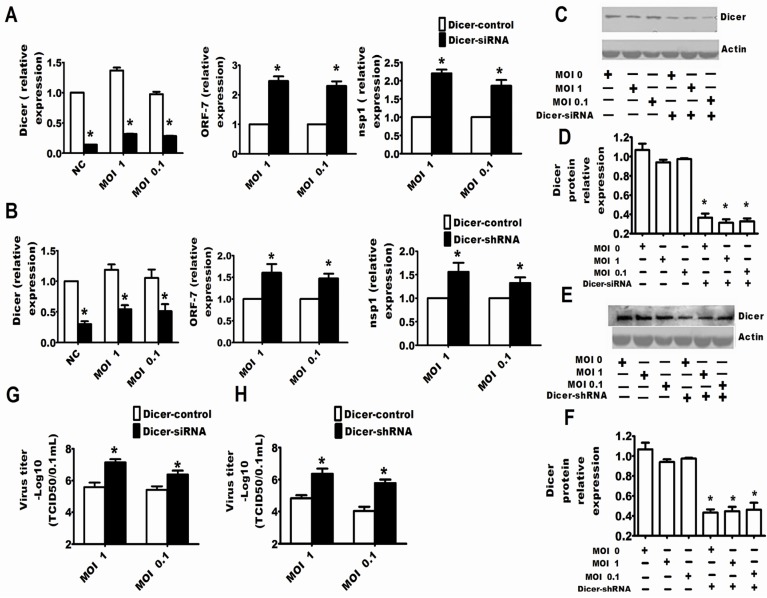
Dicer was involved in protection against the replication of PRRSV in MARC-145 cells. MARC-145 cells were transfected with Dicer-siRNA (**A**) or Dicer-shRNA (**B**). After 24 hours, the cells were infected with PRRSV at an MOI of one or 0.1. Additionally, 24 h after PRRSV infection, cells were ready for qRT-PCR of Dicer, PRRSV ORF-7 and PRRSV nsp1 (**A**,**B**) or the cells were collected for Western blots for Dicer (**C**,**E**). The results of Western blot for Dicer were quantified by Quantity One Software (**D**,**F**). The viral yields in the supernatants were quantified by a 50% tissue culture infective dose (TCID_50_) (**G**,**H**). The experiments were repeated three times. The results are from one of three independent experiments with similar observations. * *p* < 0.05.

**Figure 3:**
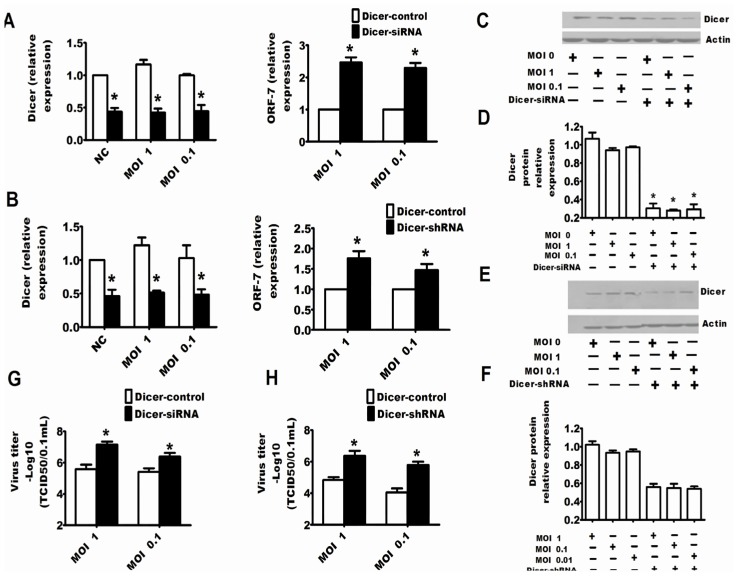
Dicer was involved in protection against the replication of PRRSV in PAMs. PAMs were transfected with Dicer-siRNA or Dicer-shRNA, and after 24 h, cells were infected with PRRSV at an MOI of one or 0.1. Additionally, 24 h after the infection of PRRSV, the cells were ready for qRT-PCR of Dicer and PRRSV ORF-7 (**A**,**B**) or the cells were collected for Western blots for Dicer (**C**,**E**). Western blot results for Dicer were quantified by Quantity One Software (**D**,**F**). Viral yields in the supernatants were also quantified by TCID_50_ (**G**,**H**). The experiments were repeated three times. The results are from one of three independent experiments with similar observations. * *p* < 0.05.

**Figure 4:**
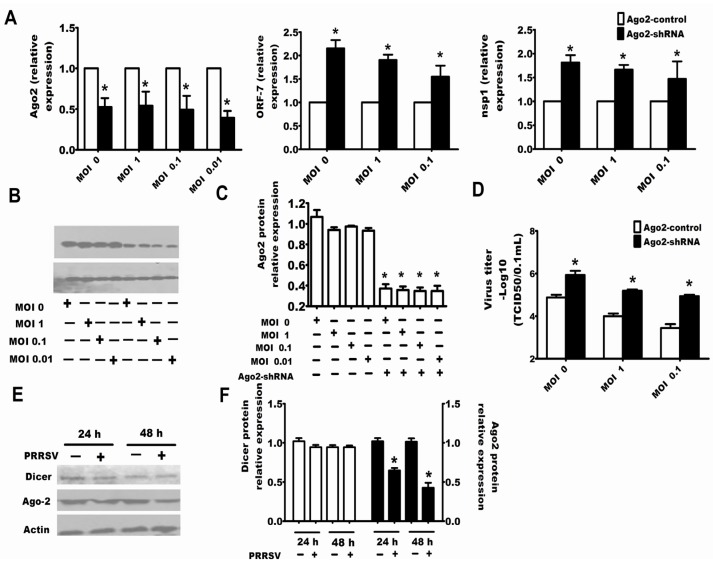
Ago-2 was involved in protection against PRRSV replication, and PRRSV downregulated the Ago-2 expression in MARC-145 cells. Cells of MARC-145 were transfected with Ago-2-shRNA (**A**), and after 24 hours, the cells were infected with PRRSV at an MOI of 1, 0.1 or 0.01. Additionally, 24 h after the infection by PRRSV, cells were collected for qRT-PCR of Ago-2, PRRSV ORF-7 and nsp1 (**A**) or cells were collected for Western blots for Ago-2 (**B**). Western blot results for Ago-2 were quantified by Quantity One Software (**C**); while the viral yields in the supernatants were also quantified by TCID_50_ (**D**); (**E**) MARC-145 cells were infected with PRRSV at an MOI of 0.1, and the cells were collected for Western blotting to detect the expression level of Dicer and Ago-2 after 24 and 48 h; and the Western blot results for Dicer and Ago-2 were quantified by Quantity One Software (**F**). The experiments were repeated three times. The results are from one of three independent experiments with similar observations. * *p* < 0.05.

It is clear that PRRSV could inhibit the RNA-induced gene silencing, and conversely, it is an attractive notion that the RNA silencing may be an anti-viral response to PRRSV. In this work, specific siRNAs or shRNAs were used to reduce the expression of endogenous Dicer to address whether the RNA silencing system played an important role in regulating PRRSV replication. Through detecting the mRNA or protein expression of Dicer, it was clear that siRNAs and shRNAs could respectively reduce the expression of Dicer (Figure 2C–F and Figure 3C–F). The results in Figure 2 and Figure 3 show that downregulation of Dicer enhanced the viral titers (Figure 2G,H and Figure 3G,H) and the levels of PRRSV RNA (Figure 2A,B and Figure 3A,B) in MARC-145 cells (Figure 2) and Porcine alveolar macrophages (PAMs) (Figure 3). Next, to confirm the above results, the specific shRNA targeting Ago-2 was used in the following experiment. The results of qRT-PCR in Figure 4A and the results of Western bots in Figure 4B,C show that the shRNAs could significantly downregulate Ago-2 expression in MARC-145 cells. Meanwhile, Figure 4A,D also shows that downregulation of Ago-2 enhanced the levels of PRRSV RNA and the viral titers in MARC-145 cells, respectively.

### 2.3. Downexpression of Ago-2 Protein Induced by PRRSV

To understand the mechanism by which PRRSV inhibited RNA-induced gene silencing, it should be firstly confirmed whether PRRSV inhibited the expression of Dicer or Ago-2 [20,21,22]. The results in Figure 4E,F show that PRRSV infection downregulated the expression of Ago-2, but did not have an effect on the expression of Dicer.

### 2.4. PRRSV nsp1α and nsp11 as the Suppressors of RNA-Mediated Gene Silencing

**Figure 5:**
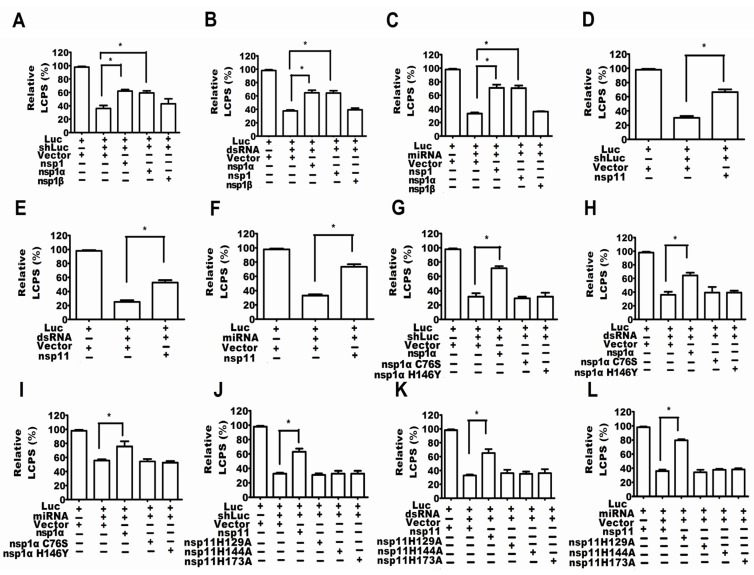
PRRSV nsp1α and nsp11 were the suppressors of RNA-mediated gene silencing (RSS). MARC-145 cells were co-transfected with firefly luciferase expression plasmid, internal control plasmid RLTK, expression plasmid of nsp1,nsp1α, nsp1β, nsp1αC76S, nsp1α H146Y, nsp11 H129A, nsp11 H144A or nsp11 K173A and firefly luciferase-specific shRNA (shLuc) (**A**,**D**,**G**,**J**), or firefly luciferase-specific dsRNA (dsRNA) (**B**,**E**,**H**,**K**), or miRNA plasmids pcDNA-miR4 and psiCHECK-UL-28 (**C**,**F**,**I**,**L**). After 48 h, the cells were ready for the dual luciferase reporter assay. The experiments were repeated three times. The results are from one of three independent experiments with similar observations. * *p* < 0.05.

To date, most discovered RSSs in animal viruses have also been interferon antagonists [5], and previous studies have shown that nsp1 and nsp11 of PRRSV were antagonists against interferon (IFN)β [23,24,25,26], so the present work selected PRRSV nsp1 and nsp11 as the candidate proteins for searching for the RSSs of PRRSV. MARC-145 cells were co-transfected with firefly luciferase expression plasmid, internal control plasmid RLTK and shLuc, or dsRNA, or miRNA. After 48 hours, the cells were ready for the dual luciferase reporter assay. The results of Figure 5A–F show that PRRSV nsp1 and nsp11 were the suppressors of RNA-mediated gene silencing (RSS).

nsp1α and nsp1β were from the auto-cleaving of PRRSV nsp1, and we found that only nsp1α was the interferon-β antagonist [27]. Consistent with the above results in this work, only nsp1α was the RSS, but nsp1β was not (Figure 5A–C).

### 2.5. Activity of Papain-Like Cysteine Proteinase Was Essential for nsp1α as an RSS

nsp1α possessed a papain-like cysteine proteinase α (PCPα) domain with a catalytic dyad [28,29] Cys-76 and His-144 were the catalytic Cys and His residues of PCPα [30]. Substitution of Cys with Ser (nsp1αC76S) or substitution of His with Ala (H146A) inactivated the activity of PCPα [30]. Our mutational study has demonstrated that the activity of PCPα was responsible for nsp1α suppressing the production of IFN-β [27], so this work also investigated whether the activity of PCPα was essential for the RSS activity of nsp1α. The results of Figure 5G–I show that all of the mutants, nsp1αC76S and nsp1αH146A, were unable to suppress the RNA silencing.

### 2.6. The Endonuclease Activity Essential for nsp11 as a RSS

PRRSV nsp11 possessed the activity of nidovirus uridylate-specific endoribonuclease (NendoU) [31] , which is a major characteristic of *Nidoviruses* [32]. From the view of bioinformatics, the NendoU domain is conserved and belongs to a small protein family whose cellular branch was prototyped by XendoU, a *Xenopus laevis* endoribonuclease [31,33]. Additionally, the active sites of NendoU are two histidines and a lysine, revealed by the crystal structures [34]. Additionally, mutating one of the three amino acids could abolish the NendoU activity [32]. Furthermore, the active sites of PRRSV NendoU should be His-129, His-144 and Lys-173 for BJ-4 PRRSV after aligning the sequences of the proteins SARS-CoV nsp15, EAV nsp11 and BJ-4 PRRSV nsp11 [31].

Our previous study showed that mutating one of the three amino acids His-129, His-144 and Lys-173 (nsp11 H129A, nsp11H144A and nsp11K173A) made nsp11 not able to inhibit the production of IFN-β [23], and this finding indicated that the endoribonuclease activity of nsp11 was necessary for nsp11 as the antagonists to the production of IFN-β.

This work further addressed whether the endoribonuclease activity of nsp11 was required for nsp11 as an RSS. The results in Figure 5J–L showed that all of the mutants, nsp11 H129A, nsp11H144A and nsp11K173A, were unable to suppress the RNA silencing.

### 2.7. The Endonuclease Activity of nsp11 and the Activity of *PCPα* of nsp1α Were Essential for nsp11 and nsp1α to Downregulate Ago-2 Protein

**Figure 6:**
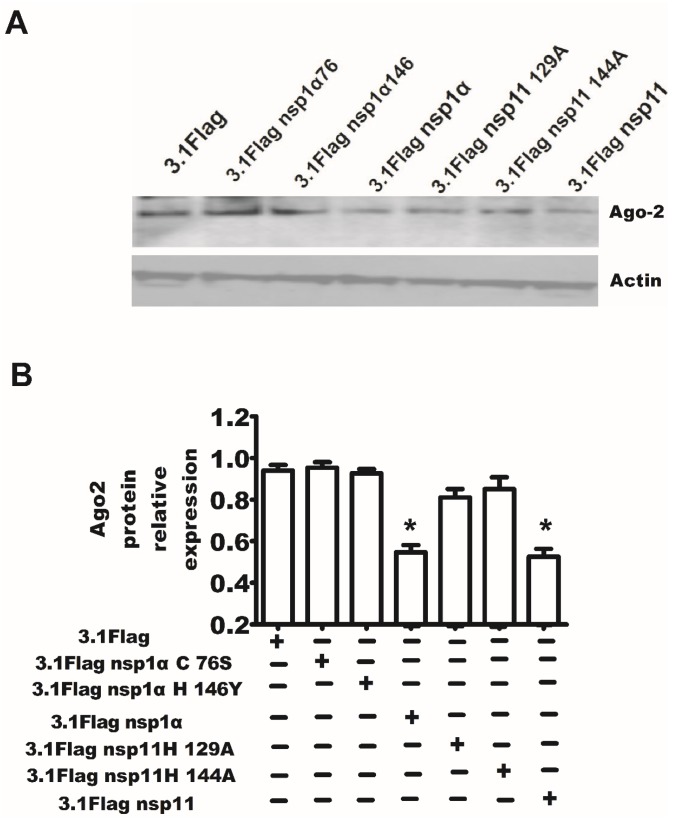
The papain-like cysteine proteinase activity of nsp1α and the activity of endoribonuclease of nsp11 were essential for them to downregulate the Ago-2 protein. (**A**) MARC-145 cells were transfected with the expression plasmid of nsp1α, nsp1αC76S, nsp1αH146A, nsp11, nsp11 H129A, nsp11H144A or control plasmid pcDNA 3.1-Flag. Additionally, after 48 h, the cells were ready for Western blotting. The Western blot results were quantified by Quantity One Software (**B**). The results are from one of three independent experiments with similar observations. * *p* < 0.05.

MARC-145 cells were transfected with the expression plasmid of nsp1α, nsp1αC76S, nsp1αH146A, nsp11, nsp11 H129A, nsp11H144A or control plasmid pcDNA3.1-Flag. Additionally, after 48 h, the cells were ready for Western blotting. The results in Figure 6 show that the endonuclease activity of nsp11 and the activity of papain-like cysteine proteinase of nsp1α were necessary for nsp11 and nsp1α to downregulate the Ago-2 protein, respectively.

## 3. Discussion

It was clear that RNA silencing could be used as a natural antiviral immunity in plants, insects and lower invertebrates [6]. The virus genome could induce an RNAi-based antiviral response, since the viral genome could form a double-stranded RNA intermediate, local secondary structures and overlapping virus transcripts during viral replication. It is worth debating whether the RNA silencing also plays an important role in controlling animal virus, and more and more evidence indicates that RNA silencing is also a part of the innate immunity of animals to the virus [5,10]. For example, Chinnappan *et al.* [9] have found that both downregulation of Dicer and Ago-2 enhanced HBV replication, and the viral titers of HBV were negatively correlated with the mRNA levels of Dicer and Ago-2; in a further study, it was found that the HBx protein was the important RSS of HBV. Maillard *et al.* [11] showed that ablating a Nodamura virus (NoV)-encoded RSS could deregulate viral replication, while a Dicer-deficient mouse cell line could rescue this mutant NoV and enhanced NoV replication.

The present work discussed for the first time the interaction between PRRSV and RNA silencing in both the MARC-145 cell line and porcine macrophages, both of which had the competent RNA silencing pathway and supported the replication of PRRSV [35]. The experimental results showed that PRRSV could suppress the RNA silencing induced by shRNA, dsRNA and miRNA (Figure 1), so it was reasonable to speculate that PRRSV modified the antiviral response of RNA silencing if the RNA silencing could be a partial anti-PRRSV response. Thereby, we tested whether the RNA silencing could be a part of the anti-PRRSV response. Because the proteins Dicer and Ago-2 are the cellular RNA silencing apparatus for shRNA, dsRNA and miRNA-induced RNA silencing, both silencing Dicer and silencing Ago-2 enhanced PRRSV replication (Figure 2, Figure 3 and Figure 4), which indicated that RNA silencing could be a defense system against PRRSV. Therefore, our present work provides new evidence that RNA silencing may be as an antiviral immunity to the invading virus in mammal cells.

Since RNA silencing is an antiviral response to PRRSV and the virus could propagate and form a persistent infection in MARC-145 cells, it is reasonable that PRRSV would encode RSS to fight against RNA silencing. Further study showed that the PRRSV nsp1α and nsp11 were the RSSs because they can suppress the RNA silencing induced by shRNA, dsRNA or miRNA (Figure 5). All of the above results indicated that although the RNA silencing could be the antiviral response to PRRSV, the virus has evolved a mechanism by which PRRSV encoded RSSs to inhibit this antiviral response in MARC-145 cells.

Ago-2 is an important protein in RNA silencing, that is, the siRNA/miRNA duplex interacts with Ago-2, which incorporates one RNA strand to form the RNA-induced silencing complex (RISC). Our present work found that PRRSV could downregulate the expression of Ago-2 (Figure 4E,F), and the nsp1α and nsp11 of PRRSV could also downregulate the expression of Ago-2 (Figure 5), which indicates that PRRSV inhibited the RNA silencing by influencing the expression of the key protein of the RNAi pathway.

Others’ and our previous studies have shown that PRRSV nsp1α and nsp11 were the antagonists of the production of IFN-β [23,24,25,26], and the activities of PCPα and endoribonuclease were respectively required for nsp1α and nsp11 as the inhibitors of the production of IFN-β [23,27]. Similar to the above results, this work not only showed that the PRRSV nsp1α and nsp11 were the RSSs, but also demonstrated that the activity of PCPα and the activity of endoribonuclease were respectively required for nsp1α and nsp11 as the RSSs (Figure 5). In fact, the relationship between the IFN pathway and the RNA silencing pathway may be an interesting topic in the future, since it suggests that the RNA silencing and the IFN pathway are overlapping. Firstly, some viral components antagonize both the RNA silencing and IFN response. For example, the most discovered RSSs were also the antagonists of type I interferon [5]. Secondly, some miRNA could be induced by IFN cytokine, and miRNA could regulate the IFN system. Thirdly, adenosine deaminase acting on the production of miRNA was also an effector of the IFN system. Therefore, the relationship between RNA silencing and the IFN system suggests that both RNA silencing and the IFN system were the first line against animal virus infections. However, the detailed mechanism of the interaction between RNA silencing and the IFN system deserves further study.

In addition, previous studies have shown that some miRNAs (miR-181, miR-23, miR-26b, *etc.*) and siRNAs that target the genome sequence of PRRSV could inhibit the replication of PRRSV [36,37,38,39,40,41]. Therefore, the present work could give a clue that the RSSs of PRRSV must be considered as another target when the siRNA, shRNA and miRNA are used as the therapy against PRRSV.

## 4. Materials and Methods

### 4.1. Animal Cell Culture and PRRSV Virus

Cells of MARC-145, the kidney cells of African green monkey from MA-104, were cultivated in Dulbecco’s Modified Eagle Medium (DMEM) (Life Technologies Corporation, New York, CA, USA) with 10% fetal bovine serum (Hyclone, Shanghai Bioleaf Biotech Co., Ltd, Shanghai, China) under a temperature of 37 °C and 5% CO_2_.

Porcine alveolar macrophages (PAMs) from the lavaged lungs of six-week-old PRRSV-negative pigs were kept in a medium of RPMI 1640 plus 10% FBS and 1% penicillin/streptomycin.

PRRSV strain BJ-4, as a gift, was kindly obtained from Yang Hanchun of China Agricultural University.

The animal study and all pig experimental procedures were authorized and supervised by the rules of animal care and use of experimental animals approved by the State Council of the People’s Republic of China [42].

### 4.2. Plasmids and siRNA

Plasmids for expressing PRRSV nsp1, nsp11, nsp11H129A, nsp11H144A, nsp11H173A, nsp1α, nsp1αC76S, nsp1αH146Y and nsp1β have been described in our previous work [23,27].

The firefly luciferase plasmid CMV-Luc for firefly luciferase expression was constructed by inserting the CMV promoter into the pGL-4.17 (Promega, Madison, WI, USA).

In dual luciferase reporter assay system (Promega), with a Renilla luciferase reporter gene, was applied as an internal control.

For the production of shRNA to silence luciferase, the forward oligonucleotides (5′-GATCCCCGAAGCGCTATGGGCTGAATACTTGAAGAGAGTATTCAGCCCATAGCGCTTCTTTTTGGAAA-3′) and the reverse oligonucleotides (5′-AGCTTTTCCAAAAAGAAGCGCTATGGGCTGAATACTCTCTTCAAGTATTCAGCCCATAGCGCTTCGGG-3′) were synthesized by Sangon Biotech, and then, the oligonucleotides were annealed to double oligodeoxynucleotides. The pSGH1-shLuc (shLuc) was constructed by using the pSGH1 vector, which can express shRNA under the H1 RNA promoter. The pSGH1 vector was digested with Bgl II and Hind III, and then, the annealed double-oligodeoxynucleotides were inserted into the digested pSGH1.

The sense sequence of siRNA for silencing of luciferase was 5′-UUGUUUUGGAGCACGGAAA (dtdt)-3′. The sequence of 5′-CCUACGCCACCAAUUUCGU (dtdt)-3′ was used as the negative control.

For the production of miR-373 expression plasmid, the miR-373 precursor sequence, the forward oligonucleotides (5′AATTCGGGATACCCAAAATGGGAGCACTTTCCCTTTTGTCTGTGCTGGGAAGTGCTTCGATTTTGGGGTGTCCCC-3′) and the reverse oligonucleotides (5′-TCGAGGGGACACCCCAAAATCGAAGCACTTCCCAGCACAGACAAAAGGGAAAGTGCTCCCATTTTGGGTATCCCG-3′) were synthesized by Sangon Biotech, and then, the oligonucleotides were annealed to double oligodeoxynucleotides and were cloned into pcDNA6.2 (Promega) to generate pcDNA6.2/miR-373 (miR-373). The 3′ UTR fragment of *Macaca mulatta* NFIB, containing the predicted target site of miR-373 [43], was generated with the primers (forward primer: 5′-CTCGAGTAATTGGAGAACCTTTCCTTCAAGC-3′ and reverse primer: 5′-GCGGCCGCCTGCGATCTACCATCAGTGAAA-3′) and was cloned into psiCHECK2 (Promega) to generate psiCHECK2/NFIB 3′ UTR (NFIB 3′ UTR).

siRNA targeting NFIB (si-NFIB) were synthesized by Genepharma (Shanghai, China), and the sense sequence of which was 5’-CCAAACUGCGCAAAGAUAUTT-3’. The sense sequence of the Negative control was 5’-UUCUCCGAACGUGUCACGUTT-3’.

For the production of shRNA to silence Dicer, the forward oligonucleotides (5′-TGCTGAATGGAAGCAGTTAGTCCCAAGTTTTGGCCACTGACTGACTTGGGACTCTGCTTCCATT-3′) and the reverse oligonucleotides (5′-CCTGAATGGAAGCAGAGTCCCAAGTCAGTCAGTGGCCAAAACTTGGGACTAACTGCTTCCATTC-3′) were synthesized by Invitrogen. Additionally, the oligonucleotides were annealed to double oligodeoxynucleotides, and then, the oligonucleotides were inserted to the shRNA-generating vector pcDNA6.2-GW/EmGFP-miR (Invitrogen, New York, NY, USA).

For constructing the plasmid to express the negative control of shDicer, the forward and reverse oligonucleotides were 5′-TGCTGAAATGTACTGCGCGTGGAGACGTTTTGGCCACTGACTGACGTCTCCACGCAGTACATTT-3′ and 5′-CCTGAAATGTACTGCGTGGAGACGTCAGTCAGTGGCCAAAACGTCTCCACGCGCAGTACATTTc-3′, respectively.

The siRNA (#6585) specific for Dicer and control siRNA (#6576) were bought from Cell Signaling Technology.

The plasmid pcDNA6.2 (Promega) and psiCHECK-2 (Promega) were the kind gifts from Liang hu Qu (Sun Yat-sen University).

The MDV_miR4 fragment was generated with the primers (forward primer: 5′-GTTGAATTCGGGCTTGTTTTGAATGTCC-3′ and reverse primer: 5′-AATGCGGCCGCTCGGCACGACAGATGTGC-3′). Digested with EcoRI and XhoI, the PCR fragment was cloned into pcDNA6.2 (pcDNA6.2- miR4). The 3′ UTR fragment of MDV-UL28 was generated with the primers (forward primer: 5′-CGGCTCGAGGGCGAAGTATTGATAGACTC-3′ and reverse primer: 5′-AATGCGGCCGCTCGGCACGACAGATGTGC-3′), and finally, the PCR product was cloned into psiCHECK-2 (psiCHECK-UL-28).

### 4.3. Antibody Used in This Study

Rabbit anti-actin IgG (Zhongshan Goldenbridge Biotechnology, Beijing, China), rabbit anti-Dicer, rabbit anti-Ago-2 and goat anti-rabbit IgG HRP-linked second antibody (Cell Signaling Technology, Boston, MA, USA) were used for Western blot experiments.

### 4.4. Experiment for Transfection and the Luciferase Assay

Sequencing analysis was used for testing the newly-prepared plasmids. Lipofectamine 2000 was used for transient transfection.

Cells were grown in 24-well plates and transfected in triplicate with CMV-Luc (pGL4.17-CMV), phRL-TK (50 ng), pSGH1-shLuc plasmid or siRNA of luciferase and/or the indicated expression plasmid. At the appointed time, PRRSV-infected cells were fetched for the luciferase assay. For the miRNA test, the above plasmids CMV-Luc and pSGH1-shLuc were replaced by the plasmids pcDNA6.2- miR4 and psiCHECK-UL-28, respectively.

### 4.5. Western Blot Experiments

The cells of MARC-145 or PAMs were grown in 24-well plates and transfected in triplicate with Dicer siRNA(100 nM) or control siRNA (100nM), and 24 h later, PRRSV (BJ-4 strain) infected the transfected cells at an MOI of 1 or 0.1; then, forty-eight hours later, the cells were dissolved in Nonidet P-40 (1%). The previous work [44] was described the detailed procedure for immunoblots. Ten percent SDS-PAGE gels were used to separate proteins. Then, the separated proteins were transferred to polyvinylidene difluoride membranes (Millipore Company, Boston, MA, USA), which were then reacted with the proper antibodies and tested by an ECL detection system (Cell Signaling Technology.

### 4.6. RT-PCR

One hundred nanomolar Dicer-siRNA or control siRNA was used in the process of transfection for the cells of MARC-145 or PAMs. Twenty-four hours later, the cells were infected with PRRSV (BJ-4 strain) at an MOI of 1 or 0.1, and 48 h later, the total RNA of the cell was extracted with TRIzol (Invitrogen, New York, NY, USA).

M-MLV reverse transcriptase was used for the PrimeScript™ RT Reagent Kit with gDNA Eraser (Takara, Dalian, China). Quantitative real-time RT-PCR (qRT-PCR) was conducted by using SYBR^®^ Premix Ex Taq™ (Takara, Dalian, China) and the 7500 First real-time PCR system (Applied Biosystems, Foster City, CA, USA). Glyceraldehyde-3-phosphate dehydrogenase (GAPDH) was used as an endogenous control. All of the samples were analyzed in triplicates. The 2^−ΔΔ*C*t^-method was applied to analyze the relative amount of target gene expression.

Table 1 shows the primers used in qRT-PCR for Dicer, ORF-7, nsp1 and GAPDH.

**Table 1:** Primers for PCR.

Primers	Sequence (5′-3′)
Dicer-Forward	GCGGTCTGCCCTGGTCAACA
Dicer-Reverse	TCTCGCACAGGGGAACGGGG
ORF7-Forward	AAACCAGTCCAGAGGCAAGG
ORF7-Reverse	GCAAACTAAACTCCACAGTGTAA
nsp1-Forward	AGGGTGTTTATGGCGGAGGG
nsp1-Reverse	AACGTCCACCGGAGTGGCTC
GAPDH-Forward	TGACAACAGCCTCAAGATCG
GAPDH-Reverse	GTCTTCTGGGTGGCAGTGAT

### 4.7. Viral Titers

Cells of MARC-145 or PAMs were transfected with Dicer-siRNA (100 nM) or control siRNA (100 nM). Twenty-four hours later, the cells were infected with PRRSV (BJ-4 strain) at an MOI of 1 or 0.1, and the supernatants were frozen and thawed in three cycles and collected at 48 h. Then the viral titers were determined by 50% tissue culture infective doses (TCID_50_).

### 4.8. Statistical Analysis

The method of Student’s *t*-test was applied for statistical analysis, and the comparisons were considered statistical significant when *p* < 0.05.
